# Freestanding 3D-interconnected carbon nanofibers as high-performance transducers in miniaturized electrochemical sensors

**DOI:** 10.1007/s00604-022-05492-2

**Published:** 2022-10-18

**Authors:** Antonia Perju, Antje J. Baeumner, Nongnoot Wongkaew

**Affiliations:** Institute of Analytical Chemistry, Chemo- and Biosensors, University of Regensburg, 93053 Regensburg, Germany

**Keywords:** Carbon nanofibers, Miniaturized electrochemical systems, Anodic stripping voltammetry, Point-of-need devices

## Abstract

3D-carbon nanomaterials have proven to be high-performance transducers in electrochemical sensors but their integration into miniaturized devices is challenging. Herein, we develop printable freestanding laser-induced carbon nanofibers (f-LCNFs) with outstanding analytical performance that furthermore can easily allow such miniaturization through a paper-based microfluidic strategy. The f-LCNF electrodes were generated from electrospun polyimide nanofibers and one-step laser carbonization. A three-electrode system made of f-LCNFs exhibited a limit of detection (LOD) as low as 1 nM (S/N = 8) for anodic stripping analysis of silver ions, exhibiting the peak at ca. 100 mV *vs* f-LCNFs RE, without the need of stirring. The as-described system was implemented in miniaturized devices via wax-based printing, in which their electroanalytical performance was characterized for both outer- and inner-sphere redox markers and then applied to the detection of dopamine (the peak appeared at ca. 200 mV *vs* f-LCNFs RE) with a remarkable LOD of 55 pM. When modified with Nafion, the f-LCNFs were highly selective to dopamine even against high concentrations of uric and ascorbic acids. Especially the integration into closed microfluidic systems highlights the strength 3D porous structures provides excellent analytical performance paving the way for their translation to affordable lab-on-a-chip devices where mass-production capability, unsophisticated fabrication techniques, transfer-free, and customized electrode designs can be realized.

**Graphical abstract:**

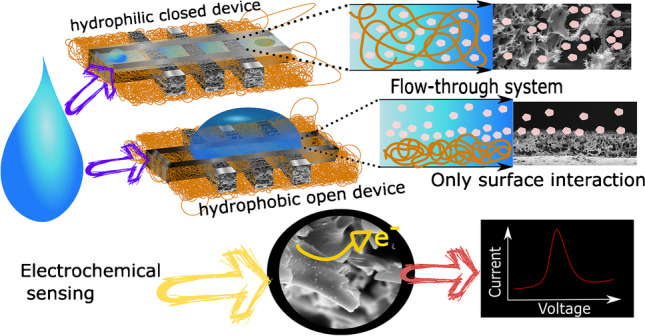

**Supplementary Information:**

The online version contains supplementary material available at 10.1007/s00604-022-05492-2.

## Introduction

Three-dimension (3D) carbon nanomaterials, e.g., graphene foam and carbon nanofibers, possess intrinsic beneficial architectures, besides their favorable electrical conductivity and low material costs, that enable their potential use as a high-performance transducer in miniaturized electrochemical sensors [1]. In comparison to 2D-based graphene, the 3D architecture not only prevents the agglomeration of graphene sheets but also promotes greater interaction between analytes and the sensing interface. Additionally, chemical- or bio-functionalities, e.g., metal nanocatalysts or bioreceptors, respectively, can be facilely and uniformly incorporated into the material, promoting their applicability in chemo- and biosensors-based electrochemical detection methods.

Various approaches have been reported for the fabrication of 3D graphene composites. Template-based methods are known as an effective strategy to generate 3D graphene with well-controlled morphologies. However, the methods suffer from high production cost and inherent complexity, in particular the need for template removal. Template-free based strategies such as hydrothermal technique [2], freeze-drying [3], and electrochemical deposition [4] are much simpler and less expensive, but the integration of the as-fabricated 3D graphene to the miniaturized electrochemical sensor is still an issue, in particular, in terms of mass-production feasibility. Even though 3D-printing of carbon materials promises high throughput manufacturing of electrochemical transducers with convenience, fast and customizable shapes, and designs, the dimensions of electrodes are typically limited to the millimeter range unless sophisticated instrumentation is applied. In addition, surface activation with harsh conditions is needed as the carbon materials contain insulating polymers, e.g., conductive graphene-doped polylactic acid filaments [5].

Despite their high-performance capability, 3D-carbon nanomaterials suffer from difficulties in integrating into microfluidic analytical systems due to their irregular shape, high thickness (in micrometers to millimeters range), and rough surface. A few studies attempted such integration, for example, graphene foam was manually inserted into a polydimethylsiloxane (PDMS) microfluidic channel [6]. Ali et al. filled photopolymers into graphene foam, and later selectively polymerized channel barriers prior to removing unpolymerized polymer with solvent [7]. Alternatively, a gel was filled into the 3D-PDMS porous scaffold before assembly and later removed by hot water [8]. The aforementioned methods are laborious, incompatible for mass-production, and prone to having poor batch-to-batch reproducibility.

Electrospun carbon nanofibers (eCNFs) make up their 3D structure via fibers with diameters in the range of a few hundreds of nanometers. Such eCNFs possess disordered graphitic structures exposing a number of edge sites that promote efficient electron transfer of electroactive species [9]. Nanofiber precursors are typically electrospun and heat-treated in a strictly controlled condition. Apart from tunable morphological/chemical characteristics of electrospun fiber precursors prior to carbonization, the production is scalable and cost-effective. Previous studies in our group demonstrated the successful generation of eCNF electrodes from electrospun polyimide (PI) nanofibers through CO_2_ laser pyrolysis, namely laser-induced carbon nanofibers (LCNFs), with great flexibility in shapes and designs. Excellent electroanalytical performances were achieved apart from fulfilling all requirements stated above. In situ generation of metal oxide decorated LCNFs was recently demonstrated with highly competitive performance for glucose sensing [10]. However, in those cases, indium tin oxide (ITO) was used as a base substrate, hindering the generation of complete 2- or 3-electrode system configurations due to short circuits via conductive film (or other conducting collectors). Even though peeling of fiber mats from the surface of the conductive collector is feasible, the resulting fiber mat’s fluffiness and uneven matt thickness make reliable laser carbonization impossible.

**Scheme 1:**
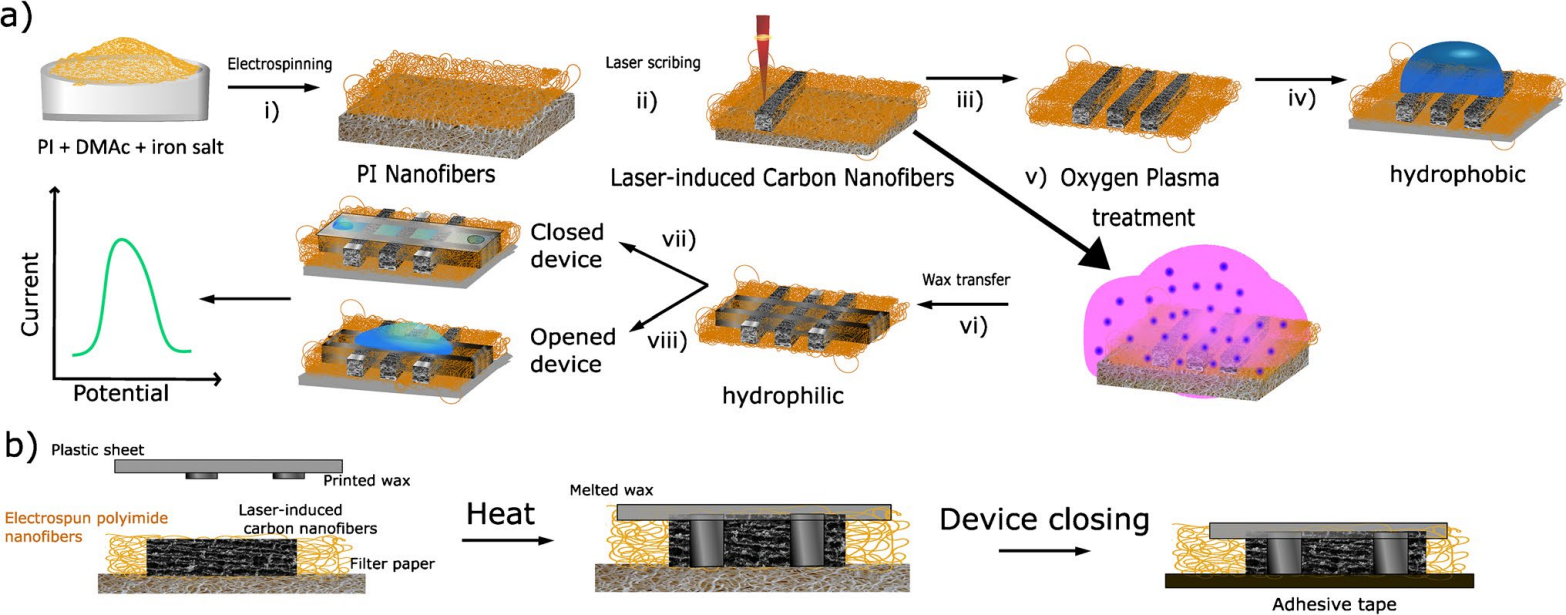
**a** Fabrication of 3D-freestanding LCNF electrodes and their integration into a device. (i) electrospinning of PI solution into precursor nanofibers collected on an oxygen plasma treated filter paper, (ii) carbonizing PI nanofiber substrate, (iii) releasing LCNF electrodes from the filter paper, (iv) the device without any further treatment (hydrophobic-open device), (v) increasing the wettability of the device via plasma treatment, (vi) creating channel wax barrier and device assembling, (vii) the device with plastic cover consisting of the wax channel (hydrophilic-closed device), and (viii) the device after the removal of the plastic cover (hydrophilic-open device). **b** Transferring of printed wax (step vi) onto the PI carried LCNF nanofiber substrate. The whole substrate piece and printed wax were sandwiched between two glass slides and placed on a heat plate (100 °C) until the wax is completely melted (~ 60 s). The filter paper was afterwards removed before closing the channel with double-sided adhesive tape (step vii). A plastic sheet can be also added to another side of the tape, enabling a robust device and ease of handling. The plastic cover will be removed in case of opened device (step viii)

In this study, we, therefore, developed freestanding LCNFs (f-LCNFs) by a one-step laser exposure as well as a robust strategy to implement them into the miniaturized electrochemical device with an integrated microfluidic channel (Scheme 1). Herein, instead of collecting PI nanofibers on an ITO sheet, a non-conductive porous filter paper was used. The conditions in electrospinning and lasing process that render f-LCNFs with favorable morphologies were initially investigated. To demonstrate the analytical performance, the 3-electrode system made of f-LCNFs without assembly into the microfluidic devices was used for anodic stripping analysis of silver. Further integration into a microfluidic analytical system was realized through the transferred wax barriers. The electroanalytical performance of the f-LCNFs-integrated miniaturized system was assessed and compared with unenclosed devices through common redox markers, e.g., ruthenium hexamine (RuHex), ferri/ferrocyanide, and dopamine. Furthermore, a highly sensitive and selective f-LCNF device for dopamine detection realized from the as-developed device was demonstrated.

## Experimental

### Chemicals and reagents

A solvent-soluble polyimide (Matrimid 5218) was purchased from Huntsman Advanced Materials (Europe) BVBA Belgium. Iron (III) acetylacetonate (Fe(acac)_3_), nitric acid (≥ 65%) (HNO_3_), dopamine hydrochloride, hexaamineruthenium (III) chloride (RuHex), potassium ferricyanide (K_3_[Fe(CN)_6_]), and potassium ferrocyanide (K_4_[Fe(CN)_6_]) were purchased from Sigma Aldrich, Germany. N, N-dimethylacetamide (DMAc) and potassium nitrate (KNO_3_) were purchased from Merck KGaA Darmstadt, Germany. Silver nitrate (AgNO_3_) was purchased from Carl Roth GmbH Karlsruhe, Germany. Silver paint was purchased from Busch GmbH & Co. KG Viernheim, Germany. Filter paper MN616 with 90 mm in diameter and 200 µm in thickness (Ref. 432,009) was purchased from Machery-Nagel Düren, Germany. Double-sided adhesive tape was purchased from tesa. Plastic sheets in A4-size were purchased from Exponent as Laser Films. Dropsens electrodes were purchased from Metrohm, Germany.

### Fabrication of PI nanofibers via electrospinning

The PI nanofibers were fabricated by electrospinning the solution containing 15% (w/v) Matrimid 5218 (750 mg) and 5% Fe(acac)_3_ (37.5 mg) (unless stated otherwise) in 5 mL DMAc. In order to obtain a homogenous mixture, the solution is sonicated for 30 min prior to stirring overnight at room temperature. The solution could be used 1 to 5 days after the preparation without significant changes in nanofiber properties.

An electrospinning device (Spraybase®) connected to a syringe pump was used to generate PI nanofibers. Here, the solution was injected into the system by a 5-mL-glass syringe connected to a polytetrafluoroethylene (PTFE) tubing where a metal needle (inner diameter of 1.2 mm) was placed at the end. The distance between the collector and the needle was set to 15 cm. The flowing rate was chosen at 10 µL/min. Environmental conditions such as humidity and temperature play an important role in the electrospinning process. Therefore, the applied voltage, between the needle and the collector, has to be adjusted according to the two parameters. This can be usually changed from 13 to 17 kV. However, the optimum conditions for spinning the nanofiber were found to be at 20 °C and around 40% relative humidity where the optimum applied voltage was ca. 16 kV.

The nanofibers were deposited on a filter paper (9 cm in diameter with a collecting area of 6 cm × 6.5 cm) that was fixed on a metal collector. Prior to collecting the fibers, the filter paper was treated with O_2_ plasma at 100 W (unless stated otherwise) to promote strong adhesion between the nanofibers and the paper’s surface.

### Laser-induced carbonization of electrospun PI nanofibers

A CO_2_ laser with 10.6 µm wavelength from Universal Laser Systems, Polytech System GmbH was used to carbonize the nanofiber substrate. Its maximum power is 30 W, and its maximum speed is 1270 mm/s. The laser process was always set to 1000 DPI and 500 PPI. For the carbonization of the nanofibers, the 2-inch lens is used. The design of the electrodes was created using CorelDRAW. The nanofiber mat is first fixed in place with tape in the laser chamber to prevent the uncontrolled movement of the mat during the scribing process. The laser conditions were varied concerning laser power and speed. The optimum conditions were found to be 4% power and 80% speed for the chosen fiber collection time. If the fiber collection time is varied, the laser parameters must be adjusted accordingly. Each electrode is scribed separately.

### Electrochemical characterization

The potentiostat PalmSens4 (the Netherlands) was employed for the electrochemical measurements. One electrochemical cell is fabricated containing a three-electrode setup, all of them are made from LCNFs. For detecting silver, anodic stripping voltammetry (ASV) was used. ASV consists of two main steps, including a deposition step and a stripping step. In the first step, silver is cathodically electrodeposited onto the surface of the working electrode by applying a constant negative potential. Then using linear sweep voltammetry (LSV) silver is stripped away from the surface by re-oxidation in the second step. Electroactive surface area (ESA) is estimated by the Randles–Sevcik equation shown below at the scan rate of 25 to 250 mV/s.

$${I}_{p}=(2.69\times {10}^{5}){n}^\frac{3}{2}AC{D}^\frac{1}{2}{\upnu }^\frac{1}{2}$$

where *I*_*p*_ is the peak current (mA), *A* is the ESA of the electrode investigated (cm^2^), *D* is the diffusion coefficient of ferricyanide (7 × 10^−6^ cm^2^/s), of RuHex (9.1 × 10^−6^ cm^2^/s), and of dopamine (6.74 × 10^−6^ cm^2^/s) [11], *n* is the number of electrons transferred in the redox reaction, *C* is the concentration of the analyte in the bulk solution (M), and *ν* is the scan rate (V/s). The value for the ESA is calculated from the slope of the *I*_*p*_ versus *ν*^1/2^ linear regression equation. Dopamine was detected via LSV. The resistance was measured with a four-point probe, containing a current source, Keithley 6430 SUB-FEMTOAMP REMOTE SourceMeter, and a Keithley Multimeter 2000 to measure the voltage between the inner two probes.

For open devices without plasma treatment, a larger volume (approx. 50 μL) of solution is required to cover the three-electrode strips while 10–30 μL is sufficient for the open and closed devices with plasma treatment in which 10 μL was used for these cases especially when f-LCNFs were employed as a reference electrode (RE). However, in case of using an Ag/AgCl external RE, 30 μL of sample was dropped on the plasma treated electrodes where the tip of RE can be inserted to the drop.

### Morphological and mechanical stability characterization

The morphology of the nanofibers was characterized using a scanning electron microscope (Zeiss/LEO 1530 Germany) at 5.0 kV. Prior to imaging, the samples have been sputtered with gold for 30 s, which should create a layer of ≈ 7 nm. The confocal microscope Olympus LEXT 3D Measuring Laser Microscope OLS4000, Germany was used to determine the thickness of the mat and the side view of the device with and without wax. Images for optical measurements were taken with a light microscope (KERN optics).

Tensile strength measurements were performed using Instron 5566 testing machine. The nanofibers were electrospun for 90 min without any further modification. The filter paper was peeled off, so both PI nanofibers and PI carrying LCNFs were tested in a freestanding form.

## Results and discussion

### Fabrications of freestanding LCNFs and their characterizations

**Fig. 1:**
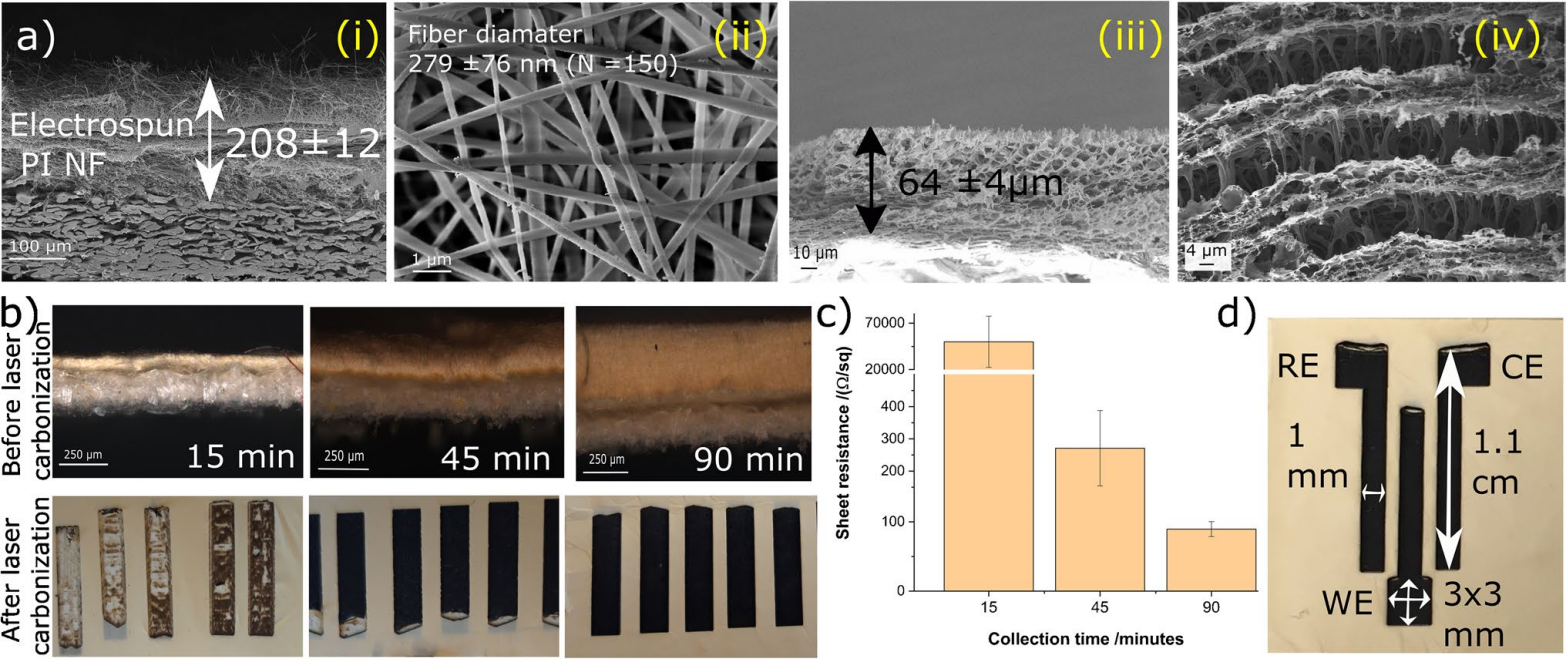
Laser-induced freestanding CNFs from electrospun PI nanofibers. **a** SEM of electrospun nanofibers/filter paper substrate (i), PI nanofibers (ii), side view of LCNFs on paper (iii), and higher magnification of LCNFs on the top view (iv). **b** Effect of electrospinning time on LCNF features, and **c** sheet resistance of the as-generated LCNFs. **d** Dimensions of working (WE), counter (CE), and reference (RE) electrodes

The collection of fiber precursors is generally performed on a conductive substrate, e.g., ITO [12], to provide a uniform electric field during electrospinning. However, this underlying surface will interfere with electroanalytical applications resulting in mixed signals from nanofiber and underlying electrodes. Research was thus performed creating a strategy for the fabrication of freestanding fibers with uniform mat thickness. A non-conductive porous substrate such as a filter paper was chosen as a sacrificial layer, since it allowed for unhindered penetration of the electric field and generated a homogeneous fiber mat thickness (Fig. 1a-i) and uniform nanofiber diameters (Fig. 1a-ii). In the end, after laser exposure, by removing the filter paper, highly porous freestanding laser-induced carbon nanofibers (f-LCNFs) 3D mats with pore sizes in a few micrometer ranges were obtained (Fig. 1a-iii and 1a-iv). Due to the roughness of the filter paper support, the f-LCNFs could easily be peeled off the support as well.

To achieve a suitable nanofiber substrate for f-LCNF generation, various parameters were studied. The effect of iron content and lasing condition was initially assessed suggesting optimal concentrations between 5 and 7% iron (Fig. S1). Furthermore, it was found that thick and dense fiber mats are needed for reliable laser-induced pyrolysis (Fig. 1b) in contrast to LCNFs made on ITO previously, which needed only 15 min collection times [10]. This implies that a conductive substrate assists with heat distribution during the laser carbonization process whereas the non-conductive substrate relies on the highly interconnected fiber network. In the end, an electrospinning time of 90 min was chosen as the electrical conductivity (156 S/m) and reproducibility of f-LCNF mats was best (Fig. 1c).

After the collection of a homogenous nanofiber mat, the pattern of the electrodes was carbonized into the mat, as shown in Scheme 1 step ii, obtaining ready to use electrodes (Fig. S2a and S2b). The fabrication strategy is not only fast (3 min for each set of electrodes (Fig. 1d) at the optimum laser speed) but also highly affordable considering the material costs of 0.01 €/electrode set (Table S1). Our current electrospinning setup allowed the fabrication of a 6.5 cm × 6 cm substrate accommodating 12 sets of three-electrode systems (Fig. S2b). A higher number of electrode sets is feasible with larger collectors or smaller electrode sizes.

Proper adhesion between electrospun nanofibers and the filter paper was studied as poor adhesion promoted bending and loose nanofibers which in turn led to unreliable surfaces for the lasing process (Fig. S3a-i to S3a-iii). Oxygen plasma treatment of the filter paper prior to fiber collection was identified as a key parameter. Calvimontes et al. demonstrated that such treatment increases surface roughness and the number of oxygenated groups of the filter paper [13], which we assume in turn assists in the attraction of fiber mat to the surface of filter paper (Fig. S3b). Finally, it was found that drops formed during electrospinning lead to defects. The agglomeration of iron in these spots could lead to substrate burning due to dramatic heat collection (Fig. S3c).

Characterizing the mechanical properties of the LCNFs, their tensile strength was measured (Fig. S4). For this purpose, nanofibers are electrospun for 90 min on filter paper, which is removed afterwards. The freestanding PI nanofibers proved to have a Young’s modulus of 20 MPa (Fig. S4d), being comparable to other reports found in literature [14]. After carbonization, the LCNF electrodes were tested again without any modification (Fig. S4b) resulting in a decreased Young’s modulus (Fig. S4d), probably caused by a more brittle nature of the laser-induced carbon. Furthermore, it was found that their obtained elasticity is similar to the skin (~ 100 kPa), suggesting the PI nanofiber carring LCNFs are suitable for developing wearable sensors in medical applications [15].

### Anodic stripping analysis of silver ions using a three-electrode system

**Fig. 2:**
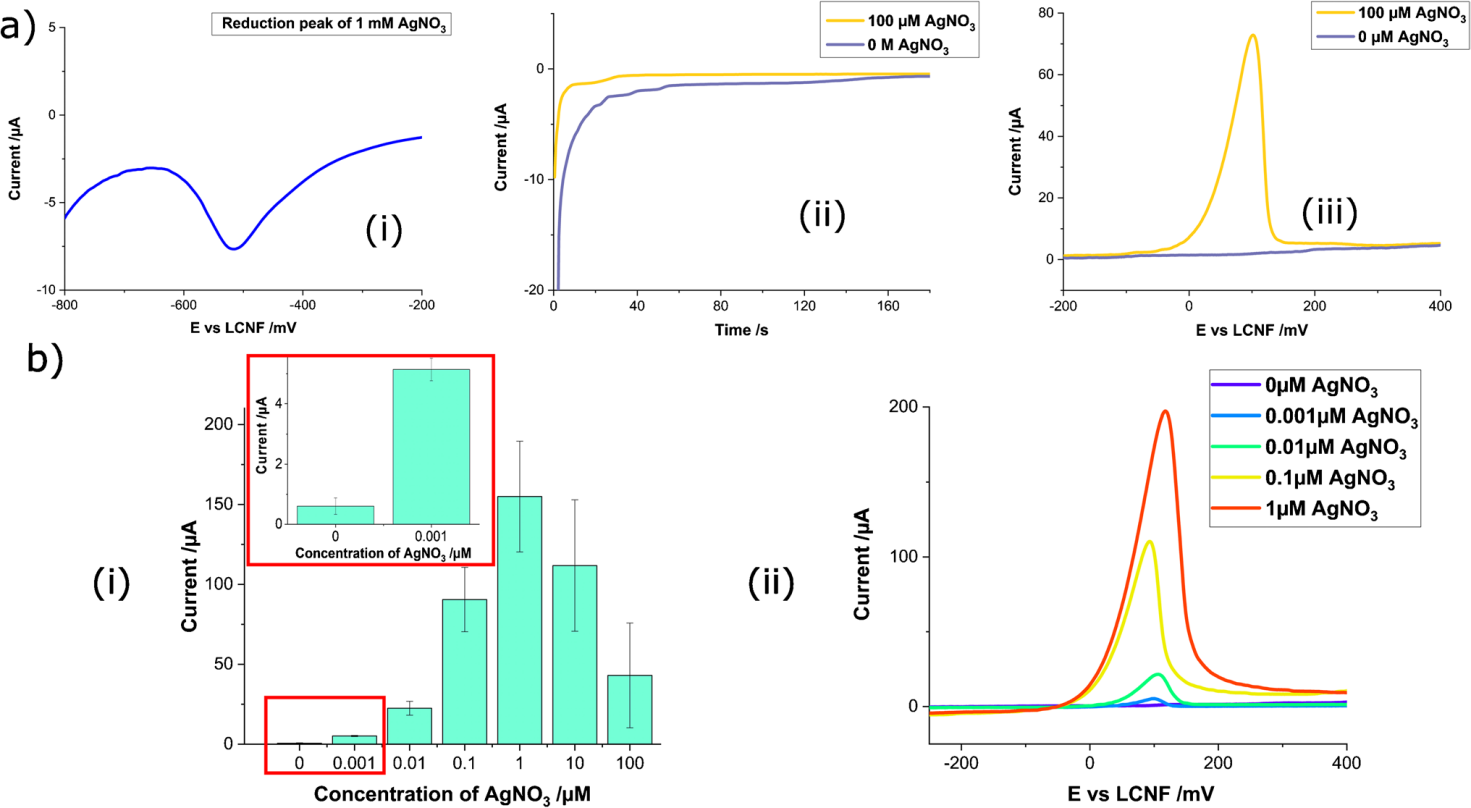
Three-electrode system of LCNFs for anodic stripping voltammetry of silver ions. **a** Cathodic peak of 1 mM silver ions (i), chronoamperograms during electrodeposition at − 600 mV (ii), and linear stripping responses in the presence and absence of silver ions (iii) with a scan rate of 50 mV/s. Silver ions were dissolved in 0.1 M HNO_3_ containing 0.1 M KNO_3_. **b** Current intensities of various silver ion concentrations (all measurements were performed in triplicate from different devices) (i), and linear sweep voltammograms of silvers in the dynamic range (ii)

To assess the electroanalytical performance of the f-LCNFs, a three-electrode system (open hydrophobic device — Scheme 1-iv and Fig. S2c) was lased and employed for ASV of silver ions. LSV scanned from − 200 to − 800 mV (*vs* f-LCNFs RE) was performed to determine the proper reduction potential of silver ions with respect to an LCNF pseudo-RE (Fig. 2a-i), suggesting − 600 mV (*vs* f-LCNFs RE) for electrodeposition of silver ions (Fig. 2a-ii). The subsequent re-oxidation by LSV scanned from − 200 to 400 mV (*vs* f-LCNFs RE) generated an anodic peak corresponding to silver at ca. 100 mV (*vs* f-LCNFs RE) (Fig. 2a-iii). These settings are similar to those published by others where Ag/AgCl and saturated calomel electrodes are used as a RE (Table 1) [16]. ASV signals increased proportionally to the silver concentration ranging from 0.001 to 1 µM (Fig. 2b). At higher concentrations, the signal drop-off is likely caused by competitive electrodeposition on the rough surface resulting in loosely bound silver grains detaching from the LCNF surface [17]. The peak shifts of the measurements seen in Fig. 2b-ii are likely due to the variation from device-to-device. Effective climate control during electrospinning as well as rotating collector could potentially reduce such variations as uniform fibers and mat thickness play a crucial role in electrode reproducibility.

**Table 1:** Comparison of electroanalytical performance of stripping analysis of silver of f-LCNF-based device to other types of carbon electrodes

Working electrode (WE)	Deposition potential *vs* reference electrode (RE)	Counter electrode (CE)	Conditions			Anodic peak of deposited silver (mV)	LOD (nM)	Ref
			*Static/stirred*	*Deposition time (min)*	*Stripping technique*			
Glassy carbon electrode (GCE)	− 500 mV *vs* saturated calomel electrode	Platinum	Stirred	10	LSV	400	5	[20]
Carbon fiber ultramicroelectrode	− 500 mV *vs* Ag/AgCl	Platinum	Static	2.5	LSV	350	1	[21]
Graphite felt (edge plane pyrolytic graphite)	− 600 mV *vs* Hg/Hg_2_SO_4_	Platinum	Static	2	LSV	− 100	10	[22]
*p*-Isopropylcalix[6]arene modified carbon paste electrode	− 250 mV *vs* Ag/AgCl	Platinum	Stirred	3	DPV	50	48	[23]
Carbon paste electrode modified with multiwalled carbon nanotubes	− 700 mV *vs* Ag/AgCl	Platinum	Stirred	0.33	DPV	100	0.74	[24]
Sulfur (S)-doped graphene (S-Gr) and a 3,3′,5,5′-tetramethylbenzidine (TMB) composite (S-Gr-TMB) modified glassy carbon (GCE)	− 100 mV *vs* Ag/AgCl	Platinum	Stirred	5	DPV	300	2150	[25]
LCNFs	− 600 mV *vs* LCNFs	LCNFs	Static	3	LSV	100	1	This work

In the end, a three-electrode f-LCNF compares favorably to other high-performance electrochemical transducers (Table 1) without the need for stirred conditions to enhance mass transport via convection [18]. The increase of mass transport can be attributed to better mixing in which chaotic nanofibrous structures of LCNFs could potentially promote efficient collisions between the analyte solution and LCNF surface. This hypothesis has been proven by our group in which PVA nanofibers possess mixing capability [19].

### Assembly of freestanding LCNF electrodes into microfluidic systems

**Fig. 3:**
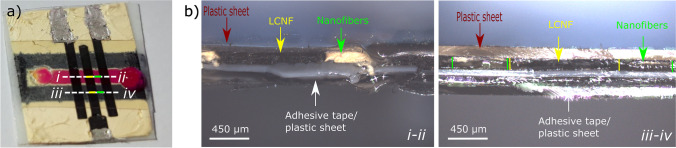
Integration of f-LCNFs in miniaturized devices. **a** Picture of one closed device with a sulforhodamine B dye in water. **b** Confocal microscope images of the side view of the device corresponded to the cross-sections shown in **a** where the left and right pictures depict the cross-sections located in the channel and at the wax barrier, respectively

The porosity, fluffiness, and electrostatics of the f-LCNFs make their assembly into microfluidic systems challenging. Here, a simple wax barrier was hence applied similar to strategies of paper-based analytical devices (PADs) [26]. Since direct printing onto the nanofiber mats was not feasible with the wax printer available, wax channels were printed onto a plastic film prior to transferring onto the LCNFs/fiber mat (Scheme 1b). After printing the wax channels, holes of 3 mm were punched into the plastic cover sheet to obtain an inlet and outlet. The wax barriers are transferred by simply putting the plastic cover sheet together with the electrodes on a heat plate, this allows the wax to melt and penetrate into the pores of the nanofibers/electrodes (Scheme 1-vi and 1b). Optimizing this process, the fiber mat thickness was investigated through fiber collection time concurrent to the variation of line width of the wax barrier (*w*_b0_) and resulting channel width (*w*_c0_) (Fig. S5a-i). Lower density mats (15 min collection time) exhibited larger wax expansion (Fig. S5a-ii), whereas denser fiber mats (≥ 30 min collection time) enabled the expansion within only 20% for all investigated *w*_b0_. The results are attributed to the amount of fiber material and void space. Consequently, a reduction by approximately 50–60% of the resultant channel was observed for the *w*_b0_ of 0.8, 1, and 1.6 mm (Fig. S5a-iii). The widest *w*_b0_ designed at 2.1 mm enabled a minimum reduction of channel width (ca. 30%) regardless of fiber density. Fluid flow and hence functionality of the resulting channels was done using sulforhodamine B (SRB) dissolved in ethanol. It was found that high-density fiber mats require a minimum amount of wax ink (determined by the designed width of the wax barrier) to completely form a hydrophobic barrier (Fig. 3a and Fig. S5a-iv).

Since aqueous solutions need to be transported within the channel system for applications in point-of-care testing (POCT), the hydrophobic LCNFs/PI nanofibers were treated with oxygen plasma (Scheme 1-v) prior to transferring the printed wax barrier (Scheme 1-vi and 1b). Such treatment dramatically changes the wettability of LCNFs and PI nanofibers (Fig. S5b-i) and can be optimized with respect to plasma power and exposure time depending on fiber mat density and thickness (Fig. S5b-ii). Accommodating f-LCNFs within the device further promotes the flow of aqueous solutions due to their inherent hydrophilicity (Fig. 3a).

Further imaging the cross-section of the assembled device revealed that the proposed fabrication strategy is highly robust, resulting in intact freestanding LCNFs and PI nanofibers (Fig. 3b-left and Fig. S6a to S6c) with a tightly sealing hydrophobic barrier (Fig. 3b-right and Fig. S6d). The available porous structures of LCNFs and PI nanofibers therefore should be ready to promote efficient mixing and overcome diffusion limitations and ultimately enhance the electroanalytical performance of the devices.

The proposed integration strategy is by far superior to other traditional sealing methods such as bonding of channels engraved in plastic sheets or using double-sided adhesive tape to create a channel barrier and later closing the channel. The irregular structure as well as the high thickness of 3D porous carbon electrodes, including LIG, make the traditional sealing methods challenging. For example, the high thickness leads to incomplete sealing between channel and electrode pieces. Moreover, pressing the channel piece over the delicate and brittle graphene flakes increases the chance of breaking the connection of working area and electrical contact pad.

### Electrochemical characterization

**Fig. 4:**
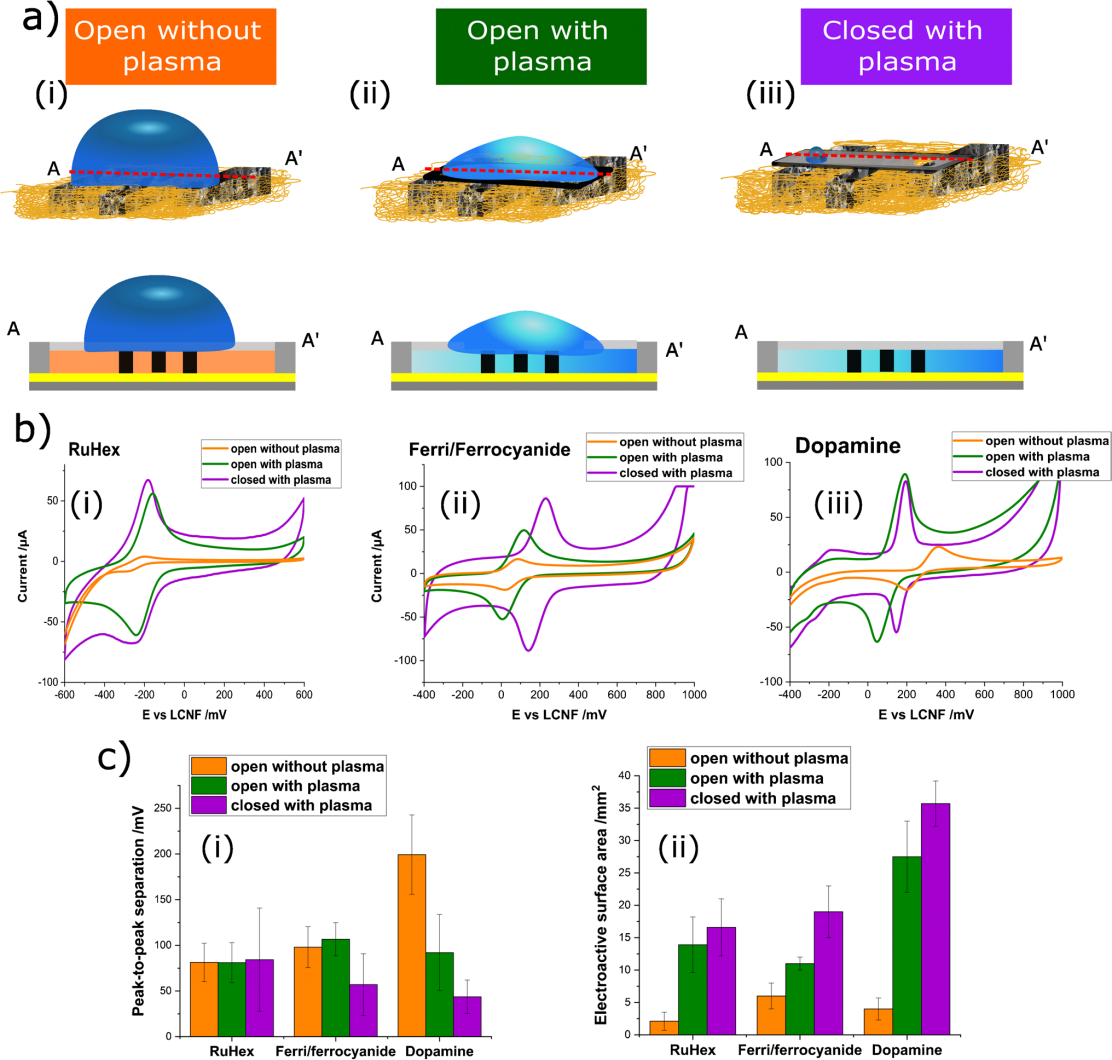
Comparison of electrochemical performance. **a** Schematic illustration of the top view and side view of an open device without oxygen plasma treatment (i), an open device with oxygen plasma treatment (ii), and a closed device with oxygen plasma treatment (iii). **b** CV’s curves of the three different configurations in presence of 1 mM RuHex (i), 1 mM ferric/ferrocyanide (ii), and 1 mM dopamine (DA) (iii) in PBS (pH 7). Scan rate: 250 mV/s. **c** Evaluation of electroanalytical performance by determining the peak-to-peak separation of CVs from 1 mM redox markers at a scan rate of 250 mV/s (i) and calculating ESA (ii) of the three configurations. All measurements were repeated using *N* ≥ 3 devices

Three sensor configurations were generated to investigate the analytical performance of the electrochemical devices (Fig. 4a). Open devices enabled electrochemical reactions taking place at their surface. Here, the exposed active surface area of the f-LCNF electrodes was different for (i) untreated- and (ii and iii) plasma-treated devices. Furthermore, closing the channel with a plastic sheet diffusion of analytes within the plasma treated porous LCNFs electrode could be achieved (iii). Outer-sphere (ruthenium hexamine (RuHex)) and inner-sphere redox systems (ferri/ferrocyanide and dopamine) [27] were investigated towards their electrochemical behaviors using cyclic voltammetry (CV) in all three setups (Fig. 4b). It was found that plasma treatment promoted proper flow inside the device and significantly increased the ESA for all investigated redox markers in comparison to the pristine LCNFs (Fig. 4b and 4c). It should be noted that the Randles–Sevcik equation cannot accurately determine the actual ESA as it is established for electrodes with non-nanomaterials or flat surfaces [28]. Since no electrochemical strategies are currently available that would allow such determination, we used the equation to merely compare active surface area resulted from different electrode treatments and device configurations. Hence, the data of ESA in this work only reflect the changing of wettability of LCNFs which subsequently affects the obtained current intensity. We do not intend to report the actual ESA gained from the as-scribed LCNFs in the present work.

RuHex as an outer-sphere redox marker is insensitive to functional groups present at carbon electrode surfaces [29]. Consequently, plasma activation did not dramatically affect the generated peak potentials and peak-to-peak separation (Δ*E*_p_) (Fig. 4b-i and 4c-i) when compared to pristine f-LCNFs. Nevertheless, the significant increase in current intensities (Fig. 4b-i) and ESA (Fig. 4c-i) of RuHex for both open and closed devices suggests that the plasma treatment only enhances the electrode’s hydrophilicity but not the electron transfer capability between RuHex and the LCNF surface. The closed channel devices do not result in a change of an electrochemical behavior of RuHex when compared to the open device configuration, emphasizing the characteristic of an outer-sphere redox marker.

Since the inner-sphere ferri/ferrocyanide is not affected by oxide and adsorption [29] treating the devices with oxygen plasma only elevated ESA but did not facilitate greater electron transfer kinetic as indicated by the comparable peak potentials and Δ*E*_p_ related to the pristine devices (Fig. 4b-ii and 4c). Interestingly, closing the channel resulted in a remarkable signal enhancement. We assume this is due to more efficient collisions at the interface compared to the open system, since the solution within the closed channel is confined inside the pores of the electrodes and thus the analyte is forced to interact with the electrode interface. The high capacitive current of the closed devices’ CV (Fig. 4b-ii) supports this assumption. As a side note, the anodic shifts of the reduction and oxidation peaks observed are likely caused by the change of LCNF pseudo-RE affected by the inner-sphere redox marker, even though it is insensitive to oxide and adsorption, which requires further investigations for elaborating such characteristic.

In the case of the inner-sphere redox marker dopamine, it can be seen that the required adsorption prior to undergoing redox reactions [29] is notably enhanced through available oxygenated groups from plasma treatment. Here, protonated dopamine (at pH 7.0) can be electrostatically adsorbed onto the LCNFs’ surface (Fig. 4b-iii and 4c-i). As the adsorption mainly contributes to the electron transfer kinetics of dopamine, no obvious change in peak potentials was observed in the closed system.

### Investigation of the reliability and stability of LCNFs as a pseudo-reference electrode

To assess the reliability of the LCNFs as an internal pseudo-RE, their cyclic voltammetric responses obtained for common redox markers were compared to that of an external RE (standard Ag/AgCl electrode). As shown in Fig. S7a, to use LCNFs as a RE results in a cathodic shift for ferro/ferricyanide, approx. 200 mV, in comparison to the CV obtained from Ag/AgCl RE which is similar to using microband carbon strip as a RE reported by Escarpa’s group [30]. However, when measuring dopamine solution, the peak potentials obtained from LCNF RE exhibited only a slight deviation in comparison to using Ag/AgCl RE (Fig. S7b). It is therefore suggested that analyte characteristics can influence the potential controlled at the working electrode which is a common behavior of pseudo-reference electrode [31]. However, considering the insignificant difference in current intensities and Δ*E*_p_ in comparison to the standard Ag/AgCl RE, LCNFs can be used as a sufficiently reliable pseudo-RE.

Further studies regarding the reliability and stability of LCNF REs over a long measuring period were conducted by determining the open-circuit potential (OCP) using standard Ag/AgCl as RE of the system, and comparing to other, commonly used REs, e.g., Ag/AgCl film of Dropsens and laser-induced graphene (LIG). As can be seen in Fig. S8, the potential controlled at the LCNFs was quite stable over the period of 3000 s similar to that of LIG and highly comparable to the pseudo Ag/AgCl on screen-printed electrode from Dropsens.

Furthermore, the stability of the three-electrode system made of LCNFs was evaluated with closed devices (*N* = 3 devices), which were cycled 30 times in 1 mM of ferri/ferrocyanide or dopamine, respectively (Fig. S9). The systems remain stable after 30 cycles for both analytes, confirming the robustness of the device. For ferri/ferrocyanide with continuous cycling, the intensity of the current is enhancing slightly due to an increase in the wettability of the electrode material. The peak couple at ca. − 250 mV and − 300 mV (*vs* f-LCNFs RE) is probably due to the redox reaction of dopamine derivative, which is commonly formed when dopamine is electrochemically measured in neutral pH [32]. Oxidation of dopamine is in this case a quasi-reversible process. Interestingly, the LCNF systems behave similarly to the gold electrodes used in [32] regarding dopamine oxidation pathways.

It can be concluded that the stability of the potential over the investigated time period is sufficient for measurements in single use point-of-care devices. Furthermore, such carbon-based, binder-free pseudo-REs open up the possibility to measure analytes dissolved in strong acidic medium such as HNO_3_ in which Ag/AgCl may encounter instability problem, i.e., the acid could dissolve the pseudo Ag/AgCl film.

### Analytical performance for dopamine sensing

**Fig. 5:**
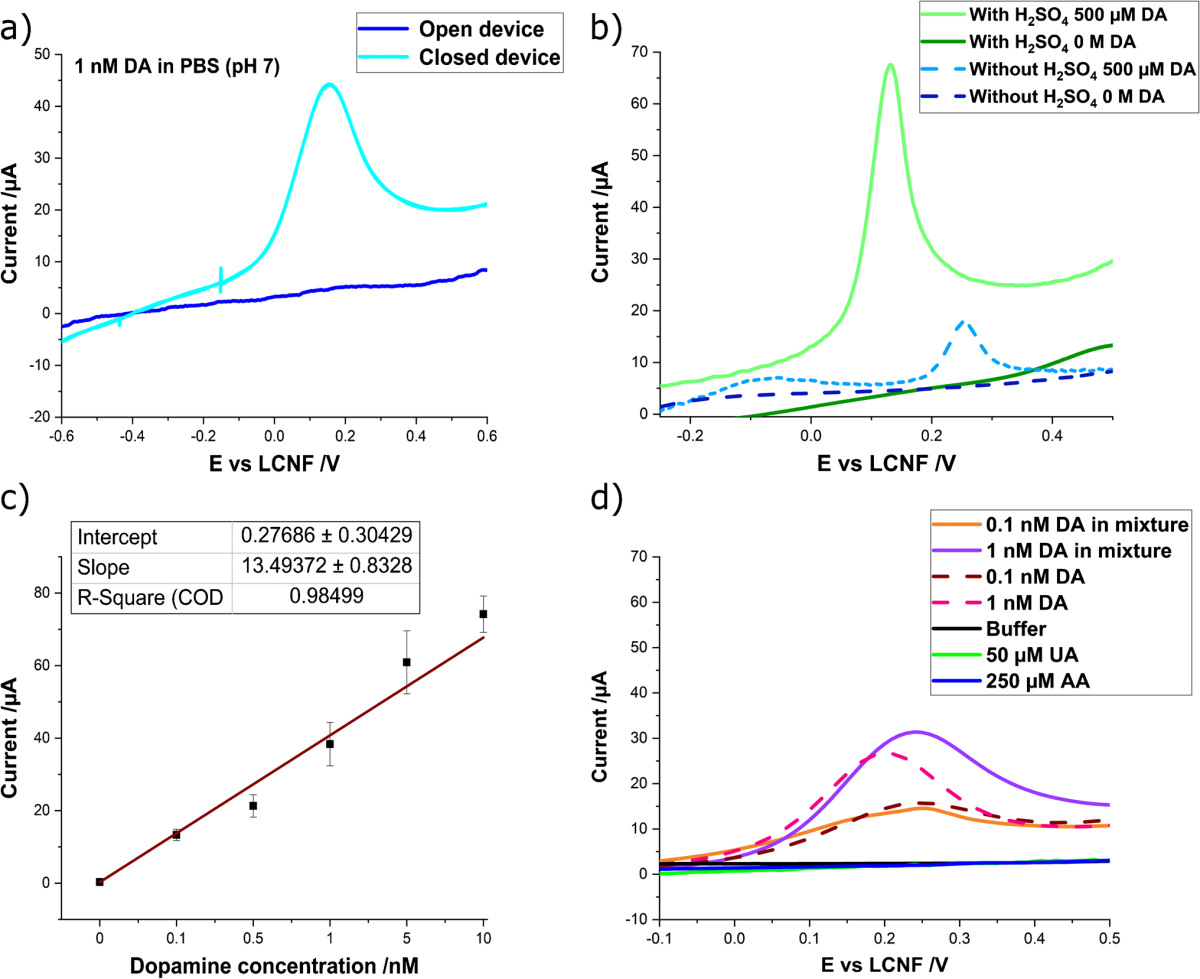
Electrochemical detection of dopamine (DA). **a** Effect of device configuration on the sensitivity of DA detection. Both devices were treated with oxygen plasma and H_2_SO_4_. **b** Sensitivity enhancement by treating the closed devices with H_2_SO_4_. **c** Calibration curve of dopamine generated from closed devices treated with H_2_SO_4_. **d** Suppression of signal interferences from uric acid (UA) and ascorbic acid (AA) by 1.25 wt% Nafion coating (closed devices with H_2_SO_4_). DA solution was prepared in PBS (pH 7). All measurements were performed by LSV with a scan rate of 100 mV/s (*N* ≥ 3 devices)

Dopamine (DA) is an important neurotransmitter involved in several neurological disorders, which requires a highly sensitive transducer to detect the alteration from normal levels (10 to 480 pM in blood) [33]. Considering the significant difference in the mechanisms supported by open and closed devices of f-LCNFs, studies were performed in both setups. No difference in electrochemical behavior for dopamine sensing was observed with open and closed devices at high dopamine concentrations (1 mM) (Fig. 4b-iii); however, the closed system was by far superior at low concentrations, i.e., 1 nM. We suggest that interactions between the analyte and interface are forced within the channel of the closed system as opposed to the open system (Fig. 5a). After modification of the electrodes with sulfuric acid (Fig. 5b), dose–response curves with high linearity over a low nM range were obtained (Fig. 5c). It should be noted that the maximum current that we could obtain from the close devices is approx. 80 μA (see also the CV of 1 mM DA in Fig. 4b-iii). This implies the maximum availability of LCNFs is reached and higher DA concentrations of 1 mM and 500 µM DA do not result in a greater current magnitude. This might be the reason why the current intensities of DA in Fig. 5a and Fig. 5b were similar even their concentrations are significantly different. The limit of detection (LOD) down to 55 pM was achieved (LOD was calculated based on 3 × STDV_blank_/slope), which renders this simple electrochemical transducer highly potent for on-site POCT dopamine detection. The calculated LOD compares favorably to similarly developed detection strategies (Table 2).

**Table 2:** Comparison of the analytical performance of most recent reports on dopamine sensing, especially focusing on using 3D-carbon nanomaterial

Materials	Linear range	Sensitivity	LOD (nM)	Ref
CVD 3D graphene foam	0–25,000 nM	619.6 μA mM^–1^ cm^–2^	25 (S/N = 5.6)	[36]
Carbon-black modified GCE	0.1–40 µM	0.61 µA µM^–1^	13	[37]
Gold nanobipyramid/multiwalled carbon nanotube hybrids	50 nM–2.7 mM	-	15 (S/N = 3)	[38]
SU-8 photoresist based pyrolytic carbon (PyC)/PyC-O_2_	50 nM–1 µM	1.2 A M^–1^ cm^–2^/2.7 A M^–1^ cm^–2^	40/20	[39]
Molybdenum (IV) disulfide nanosheets deposited on carbon nanofibers (CNFs)	0–60 µM	6.24 µA µM^–1^ cm^–2^	36 (S/N = 3)	[40]
3,4,9,10-perylene tetracarboxylic acid functionalized graphene–multiwalled carbon nanotube–gold nanoparticle nanocomposite modified glassy carbon electrode (PTCA-RGO-MWCNTs-Au NPs/GCE)	1–100 µM	0.124 µA mM^–1^	70 (S/N = 3)	[41]
3D GF/ITO	0–60 µM	1.88 µA µM^–1^ cm^2^ (0–5 µM) and 1.44 μA μM^–1^ cm^–2^ (5–60 μM)	100	[42]
ZnO nanosheet balls (ZnO NSBs) on three-dimensional graphene foam	1–80 µM	0.99 μA μM^−1^	10 (S/N = 3)	[43]
Au nanoparticles-ZnO nanocone arrays/graphene foam	0–80 µM	4.36 μA μM^−1^	40 (S/N = 3)	[44]
Freestanding graphene foam-carbon nanotube composite coupled with gold nanoparticles	0.10–48 μM	12.72 μA μM^−1^ cm^−2^	1.36 (S/N = 3)	[45]
LCNF electrodes (miniaturized systems)	0–10 nM	13.9 µA nM^–1^	0.055 (S/N = 3)	This work

A strong non-specific signal by uric (UA) and ascorbic acid (AA), common interferences for dopamine detection [34] (Fig. S10a and S10b), could be avoided by applying Nafion as a well-known cation exchanger that possesses permeability to cations [35]. Specifically, its inherent negative charge from sulfonate groups in the structure can suppress anion interferences, e.g., UA and AA, as successfully demonstrated previously [34]. At the same time, its hydrophobic molecular backbone limits its useful concentration range, i.e., Nafion coating of an f-LCNF WE with high concentration solution, e.g., 5 wt%, leads to poor wettability of the device. In the end, we found that 1.25 wt% Nafion and plasma treatment at 200 W (instead of 100 W) was optimal to facilitate proper device assembling and solution flow (Fig. S8c and d). The finally established fabrication process of f-LCNFs for dopamine detection entails (i) Nafion application (only at WE), (ii) plasma treatment, and (iii) H_2_SO_4_ incubation, the latter being performed after device assembly. As expected, the LCNFs modified with Nafion were able to suppress any signal interferences from UA and AA even at concentrations several times higher than dopamine (more than 10,000-fold the dopamine concentration) (Fig. 5d).

## Conclusion

Herein, we present laser-induced carbon nanofibers as freestanding electrodes that can be easily integrated into miniaturized devices. Ultralow detection limits for silver ions via stripping voltammetry suggest their applicability towards the detection of AgNPs use as signal generating label in (bio)sensors [46, 47], especially as analyses do not require stirring conditions due to the immense porosity of the f-LCNFs. Also, the results indicate that further modification with electrodeposited metal nanocatalysts may be of great interest in future studies especially at low metal salt concentrations. Overall, the presented strategy is robust, easy to perform, and generates electrodes at < 0.02 € per electrode. The comparison between open and closed devices supports well the beneficial feature realized by integrating 3D porous electrodes in microfluidic analytical systems. The tremendous enhancement offered by the 3D structure of the electrode has been proven in the detection of dopamine down to the pM range. Furthermore, the proposed assembling method is highly attractive for producing POC devices as it requires < 10 min (without the electrospinning) for each device and costs < 1 €/device (Table S2). This will enable flow-through applications, where the reaction takes place inside the pores of the electrodes in contrast to other microfluidic systems, where the reaction is limited to the 2D interface between the electrodes and the channel. In the end, we suggest that devices based on f-LCNF integrated into a flow system are highly advantageous for point-of-care devices as their fabrication suits the ASSURED criteria well including mass-production possibilities. Furthermore, we predict that f-LCNFs can revolutionize paper-based and wearable devices in which not only surface-bound signals (such as in optical systems and current electrochemical strategies), but also 3D signals can be generated. Through the integration of selectivity agents such as aptamers, antibodies, or chemical recognition molecules, the high specificity needed for real sample analysis will be enabled just like with other electrochemical systems.

## Supplementary Information

Below is the link to the electronic supplementary material.

Supplementary file1 (DOCX 1.66 MB)
