# Supplementary material for: Freestanding 3D-interconnected carbon nanofibers as high-performance transducers in miniaturized electrochemical sensors
Source: Mikrochim Acta. 2022 Oct 18;189(11):424. doi: 10.1007/s00604-022-05492-2 (PMC9579100; doi:10.1007/s00604-022-05492-2)
Supplement: Supplementary file 1 — Supplementary file1 (DOCX 1.66 MB) [file 604_2022_5492_MOESM1_ESM.docx]

Supporting Information

Freestanding 3D-interconnected carbon nanofibers as high-performance transducers in miniaturized electrochemical sensors

Antonia Perju,^a^ Antje J. Baeumner ^a^ and Nongnoot Wongkaew^*a^

^a^Institute of Analytical Chemistry, Chemo- and Biosensors, University of Regensburg, 93053 Regensburg, Germany

^*^Corresponding author

E-mail: [nongnoot.wongkaew@ur.de](mailto:nongnoot.wongkaew@ur.de)

ORCID: 0000-0002-6118-6182


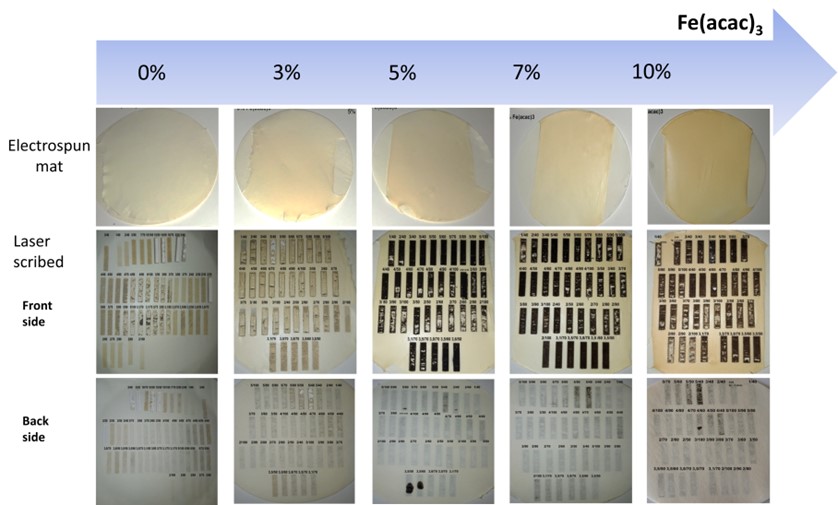


**Fig. S1 Effect of iron content and lasing conditions**. Polymer solutions were prepared by dissolving 15% (w/v) polyimide (750 mg) and different Fe(acac)_3_ contents, i.e., 0%, 3% (22.5 mg), 5% (37.5 mg), 7% (52.5 mg), and 10% (75.0 mg) in 5 mL DMAc. The electrospun nanofbers were collected for 15 minutes on filter paper. Different laser settings (% of power/%of speed) were used to carbonize the nanofibers.

The iron content in the polyimide (PI) solution was varied from 0- to 10 % (rel. to the mass of PI). The same lasing conditions were tested on all mats. As it can be observed, especially on the front side of the scribed mats, the nanofibers were burned when the iron content was absent or present in a relatively low amount, while for 5% and 7% the mats were carbonized more homogenously. This proves that the iron present in the nanofibers facilitates heat dissipation during carbonization similar to the previous work [1].

**Table S1 Cost estimation of electrode materials used for preparing electrospun nanofibers on filter paper substrate**

| **List of material** | **Price per unit** | **Amount used for each fiber mat** | **Price per mat** | **Price per each electrode set**  **(one mat contained 10 sets of electrodes)** |
| --- | --- | --- | --- | --- |
| Matrimid 5218 | 0.059 €/g | 0.1875 g | 0.01 € | 0.001 € |
| Dimethylacetamide (DMAc) | 0.0037 €/mL | 1.25 mL | 0.005 € | 0.0005 € |
| Fe(acac)_3_ | 0.15 €/g | 0.0094 g | 0.0014 € | 0.00014 € |
| Filter paper | 0.079 €/piece | 1 piece | 0.079 € | 0.0079 € |
| Total price per each electrode set (three-electrode system) | | | | **0.01 €** |

**Table S2 Cost estimation of materials used for device assembly**

| **List of material** | **Price per unit** | **Amount used for each device** | **Price per device** |
| --- | --- | --- | --- |
| Double-sided adhesive tape | 0.00238 €/cm^2^ | 6.25 cm^2^  (for device of 2.5 cm × 2.5 cm) | 0.015 € |
| Plastic sheet for wax printing | 0.0045 €/cm^2^ | 6.25 cm^2^  (for device of 2.5 cm × 2.5 cm) | 0.028 € |
| Wax ink | 2.2×10^-5^ €/cm^2^ | 0.88 cm^2^  (for rectangle channel barrier with dimension of 0.7 cm × 1.9 cm, width of barrier (*w_b0_*) of 0.2 cm) | 2 × 10^−5^ € |
| Silver paint | 0.024 €/μL | 5 μL | 0.12 € |
| LCNF electrode set (Table S1) | 0.01 €/piece | 1 piece | 0.01 € |
| Total price per each device | | | **0.17** **€** |


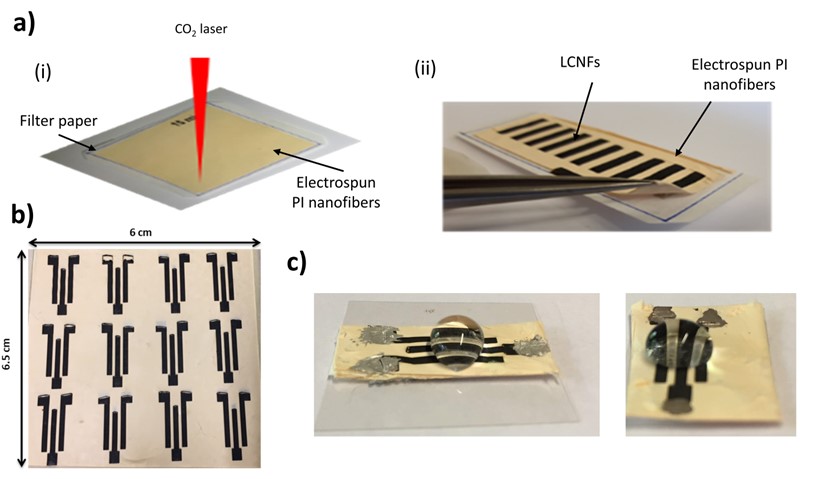


**Fig. S2 Production of laser-induced carbon nanofibers (LCNFs) using filter paper as an initial support.** a) PI nanofiber collecetd on filter paper as a substrate for laser carbonization (i), and releasing PI nanofiber mat carried LCNFs from the filter paper. b) LCNF electrodes on the substrate. c) An LCNF device after transfering to a plastic sheet. The electrical contact pads were painted with silver paste. The nanofibers were collected for 90 minutes on the filter paper.

After the nanofibers are electrospun, the mat is fixed onto the laser support and carbonized (Fig. S2a-i). After the carbonization, the nanofibers together with the LCNFs can be easily peeled off from the substrate, as seen in Fig. S2a-ii. A mat has the size of 6 x 6.5 cm as shown in Fig. S2b, which can accommodate 12 sets of three-electrode systems for the present electrode design. Afterward, the devices can be cut using a normal scissor and put on a plastic sheet with double-sided adhesive tape to ensure robustness and stability. Without any treatment the electrodes and PI nanofibers are hydrophobic, but a small volume (50 µL) can be added as shown in Fig. S2c and used for the measurements in this work.


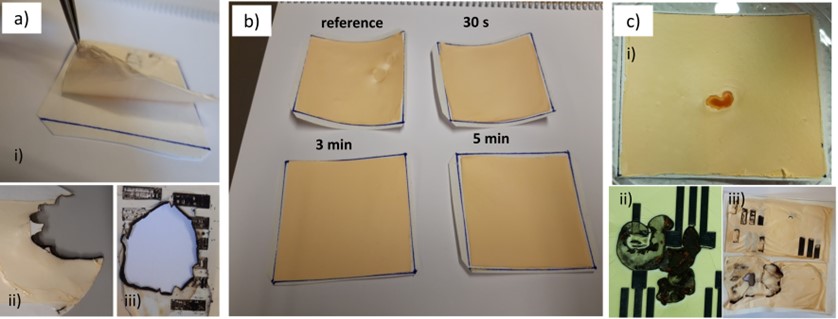


**Fig. S3 Effects of plasma-treated filter paper and defects on the production of LCNF electrode.** a) Electrospun nanofiber mat collected on a filter paper without plasma treatment (i), and the burned fiber mat after exposure to the CO_2_ laser (ii & iii). b) Electrospun nanofiber mat collected on a filter paper treated at various plasma exposure time and their comparison with the mat collected on an untreated filter paper. c) Dripping of the spinning solution on the nanofiber mat (i), causing iron aggregation and thus burning of nanofibers. Nanofibers containing 15 (w/v)% PI and 5% (rel. to the mass of PI) Fe(acac).

Adhesion of the nanofibers to the substrate plays an important role as can be seen in Fig. S3ai-iii. If the nanofibers did not adhere well, during scribing they tend to burn, deteriorating the mat and the filter paper (Fig. S3aii-iii). This is likely due to a gap of air in between the fibers and the substrate. To facilitate the adhesion of the nanofibers to the substrate, oxygen plasma was used. Prior to collecting the nanofibers, the filter paper was treated with oxygen plasma (100 W, 100% O_2_) (Fig. S3b). Without plasma treatment (denoted as reference) or with the short treatment time, e.g., 30s, the nanofibers/filter paper bends up, implying connections between fibers are stronger than fibers to the filter paper surface. When such substrate was fixed prior to laser scribing, a gap between fibers and filter paper may be created, which probably hinders the uniform heat dissipation during scribing. In contrast to the longer exposure time, e.g., 3 and 5 minutes, the substrates are flatter. The nanofibers, therefore, resulted in greater LCNF features (data not shown) where 5 minutes of plasma treatment was further used. Finally, if during the electrospinning process spinning solution has dropped on the mat (Fig. S3c-i) this causes a defect in the mat in which an agglomeration of the iron is formed. When the laser beam reaches the iron aggregate, it will carbonize also the surrounding zone uncontrollably (Fig. S3c-ii). Alternatively, it can just burn the mat similar to no adhesion of the nanofibers, shown in Fig. S3c-iii. The result also suggests that uniform heat dissipation during laser carbonization crucially requires homogeneous distribution of iron along the as-spun nanofibers.

**
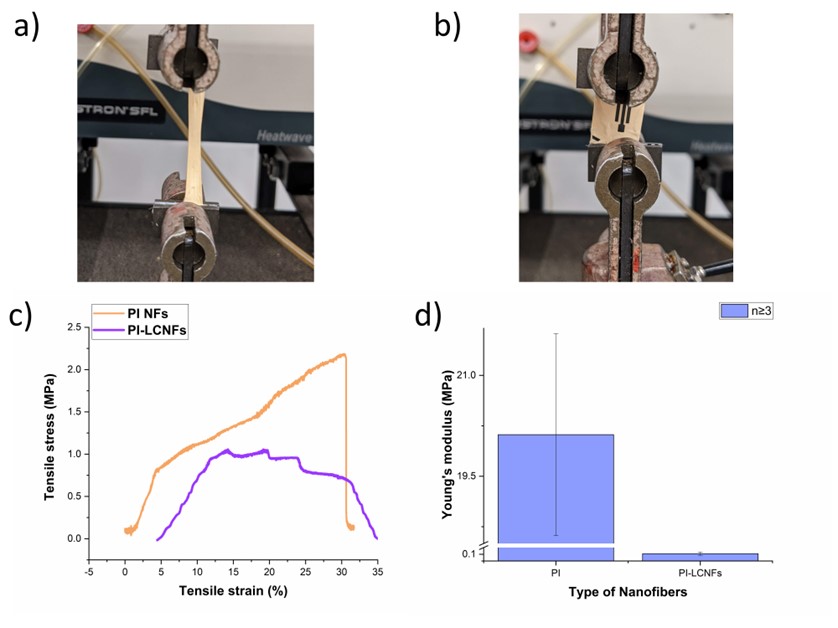
**

**Fig. S4 Study on mechanical stability.** a) The measurement setup for PI nanofiber mat (PI NFs). The mats were cut into 45 mm long and 10 mm wide strips. The filter paper was peeled off and one strip was attached to the holders on the device. The measurements were performed in triplicate. b) The PI nanofiber carried LCNFs (PI-LCNFs) were cut into 20 mm long and 15 mm wide pieces. The filter paper was peeled off and they were set up vertically in the direction of electrode strips as shown in the photograph. The measurements were performed in quadruplicate. c) The tensile stress *vs* strain curves of PI nanofiber mat and PI-LCNFs. d) Young’s modulus obtained from c). The raw data of the tensile strength were plotted in Origin and the data points outside the linear range corresponding to the elastic deformation were masked out for both PI nanofiber mat and PI-LCNFs. Young’s modulus was determined by fitting linearly the slope in the elastic region (sharp increase) in the tensile stress *vs* strain curve.

As can be seen in Fig. S4c, the PI nanofiber mats exhibit one point of fracture at the end which is a typical behavior for nanofiber mats [2, 3], while for the PI-LCNFs four different points of fracture can be observed, corresponding to the three LCNF electrode strips and nanofiber mats at last fracture point.


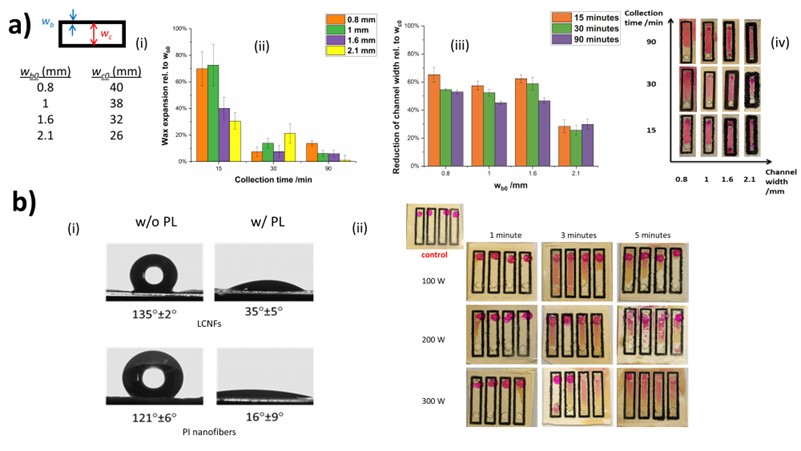


**Fig. S5 The fabrication of a microfluidic device containing PI nanofibers.** a) Influence of channel designs and fiber collection time. Channel design with various line thicknesses of wax barrier (*w_b0_*) and corresponding width of channel (*w_c0_*) (i), effect of fiber collection time on percentage of wax expansion ($\frac{w_{b}-w_{b0}}{w_{b0}}$ ×100, *w_b_* indicates the line width after wax melting) (ii), effect of *w_c0_* on percentage of channel width reduction ($\frac{w_{c0}-w_{c}}{w_{c0}}$ ×100, *w_c_* refers to the width of channel after wax melting) (iii), study of solution flow within assembled device at various fiber collection time and *w_b0_* where *w_c0_* of 20 mm was designed for the study (iv). The wax channels were created by heating the devices for 1 min at 100°C. Leaking test was performed by flowing 10 µL of solution containing sulforhodamine B (SRB) dissolved ethanol into the devices. b) Increasing wettability of LCNFs and PI nanofibers by oxygen plasma treatment. Water contact angle of LCNFs and PI nanofibers after oxygen plasma treatment for 3 min (i), and optimization of the oxygen plasma treatment regarding power and treatment time (ii). Here, SRB dissolved in water was used for leaking test.

The widths of the barriers w_b0_ and w_c0_ of the channels for the studied systems are displayed in Fig. S5a-i. The wax expansion was investigated with respect to different collection times and different barrier widths (Fig. S5a-ii). This has a great influence on the performance of the device since the size of the channel correlated directly to the surface of the electrode used in the detection reaction. For fiber collecting time of 90 mins, the least wax expansion was observed for the thicker barrier, implying that the wax melts and diffuses vertically filling the pores of the nanofibers mat through the whole thickness of the mat. Furthermore, the direct correlation of the wax expansion is the reduction of the channel width (Fig. S5b-iii). The thickest barrier preserves the best integrity of the channel. In order to ensure that there is no leaking the mats collected for different times were tested with the different barriers as can be seen in Fig. S5a-iv. For the narrow barrier, the solution leaks outside, while the thicker barrier seals the channel regardless of the mat thickness. Pristine PI nanofibers and LCNFs are hydrophobic (Fig. S5b-i). Therefore, to increase hydrophilicity of the materials enabled the flow of aqueous solution, both PI nanofibers and LCNF are treated with oxygen plasma. The dramatic decrease in contact angles proves that the oxygen plasma effectively renders both PI nanofibers and LCNFs with great wettability. To ensure proper flowing of aqueous solution inside the channel different powers and different exposure times were investigated for the plasma treatment (Fig. S5b-ii). In order to avoid leaking the best conditions were chosen to be 200W and 3 minutes.


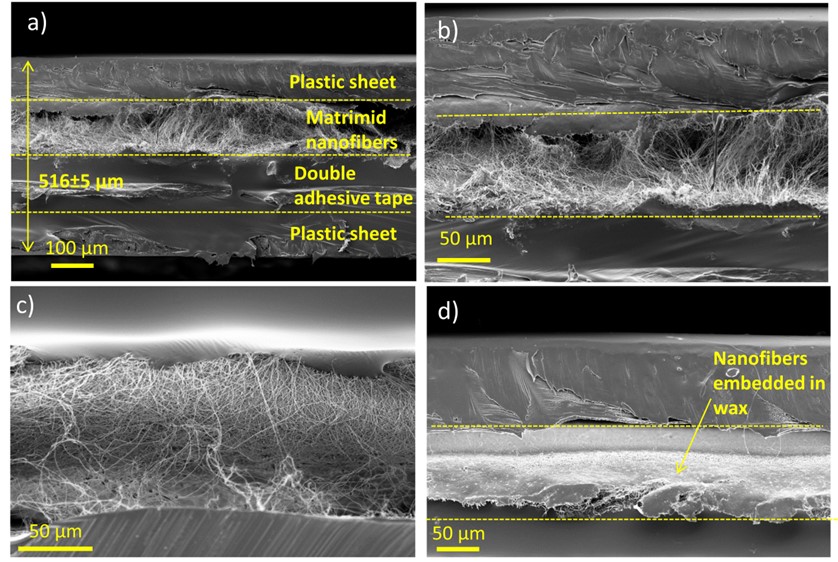


**Fig. S6 SEM images inside the microfluidic device.** a) Side view of the device showing its components at the area contained nanofibers which is located in the middle. b) and c) the side view shown in a) with higher magnification. d) Side view at the area where wax barrier is located.

As shown in Fig. S6a, the whole device has a thickness of approx. 0.5 mm. It can be observed how the plastic sheets completely seal the device. On the plastic sheet underneath the device also with glue can be observed, which is defect caused by the preparation of the sample for SEM. In Fig. S6b and S6c, the nanofibers fill the channel inside the device, creating a mesh, through which the solution can flow through. This is contrast to the area where the wax barrier is located (Fig.S6d). The mesh structure of nanofibers is filled with melted wax, therefore confining the area of the channel possible, and thus preventing the solution leaking. SEM images of LCNF embedded within the channel are not shown here because the structure was often destroyed during sample preparation for imaging.


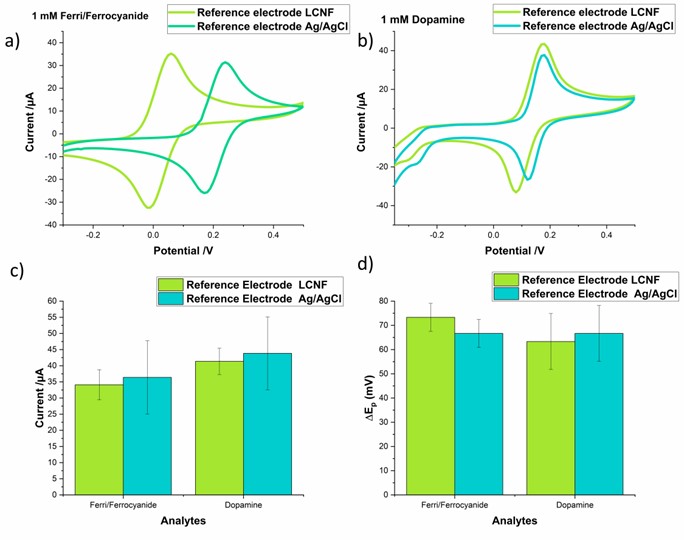


**Fig. S7** **Comparison of analytical performance between using LCNF and Ag/AgCl as a reference electrode (RE).** a) Cyclic voltammograms of 1 mM Ferri/Ferrocyanide on open device using LCNF as RE and using an external Ag/AgCl RE. b) Cyclic voltammograms of 1 mM dopamine on open device using LCNF as RE and using an external Ag/AgCl RE. c) The peak intensity was determined from the oxidation peaks of the respective voltammograms shown in a) and b). d) Peak-to-peak separation was determined from the cyclic voltammograms in a) and b). All LCNF devices were treated with oxygen plasma to increase the wettability and wax channels were applied to confine the area of the solutions. The plastic cover was removed to render open device configuration for the accessibility to an external Ag/AgCl RE. In case of using an external Ag/AgCl RE, internal LCNF electrodes were used as a counter electrode. A 10 µL drop was added on top of the devices and in the case of the external RE, the electrode was immersed in the drop. The scan rate was 100 mV/s. All measurements were performed in triplicate.

**
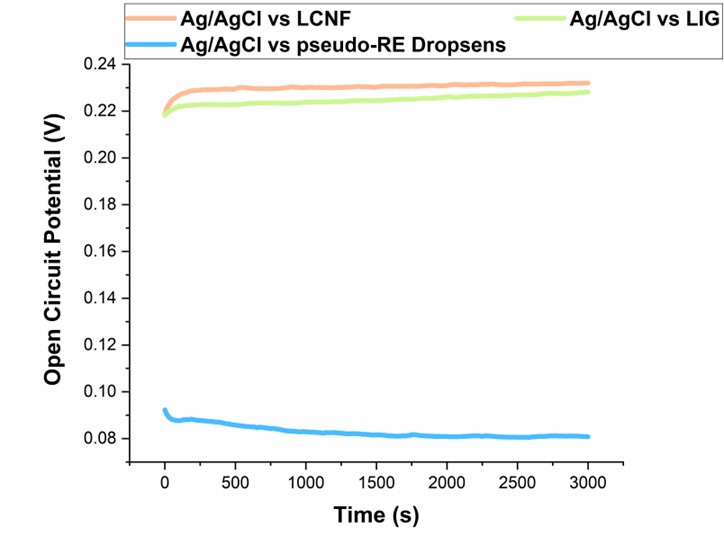
**

**Fig. S8 Investigating stability of LCNFs being used as a reference electrode (RE) and the comparison to other referencing electrode systems.** The open circuit potentials of various electrode materials against a standard Ag/AgCl RE

were measured in 1 mM ferri/ferrohexacyanide solution (PBS, pH 7.0 with 0.1 M KCl). A commercially available screen- printed Ag/AgCl film of Dropsens electrode (Metrohm) was also investigated for comparison.


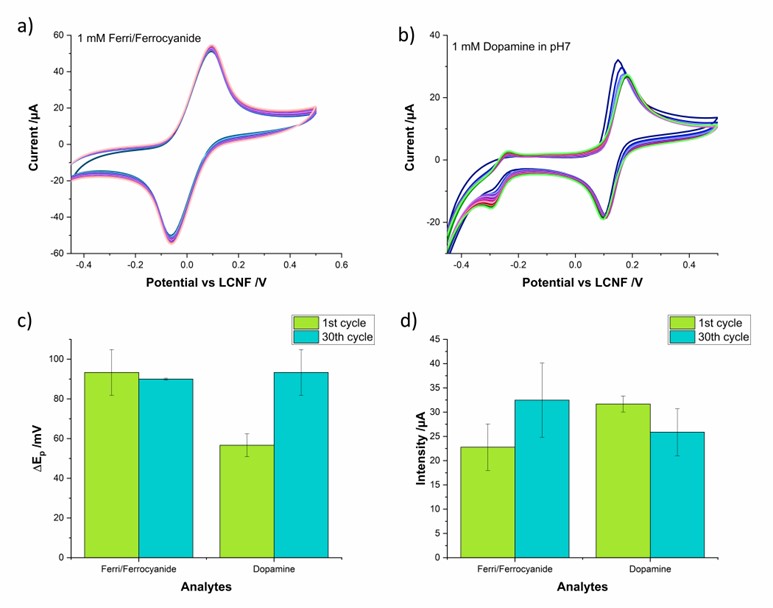


**Fig. S9 Stability of the three-electrode system in the closed device.** a) and b) Cyclic voltammograms of 1 mM Ferri/Ferrocyanide, and 1 mM dopamine, respectively, running consecutively for 30 cycles. c) Peak-to-peak separation evaluated from the first and last cycle of the voltammograms. d) Intensity of the oxidation potential for the first and last cycle. The sample volume used was 10 µL. The scan rate is 100 mv/s. The measurements were repeated on fresh electrodes. *N* = 3


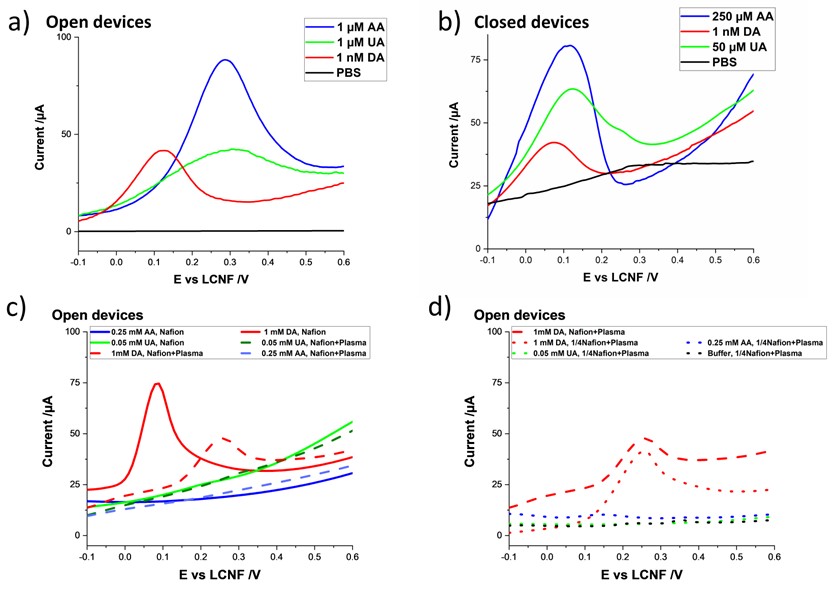


**Fig. S10** **Interference caused by uric acid (UA) and ascorbic acid (AA).** a) and b) Linear sweep voltammogram (LSV) of dopamine compared with those obtained from uric and ascorbic acids in open and closed devices, respectively. c) Eliminating interferent signals from UA and AA by drop-drying 1 μL of undiluted Nafion (5 % (w/v)) on the WE as well as the post-plasma treatment. d) Effect of Nafion dilution on LSV signals. The sample volume used was 10 µL. The scan rate is 100 mv/s.

In the open device the peak potential of uric acid (UA) and ascorbic acid (AA) is very close to the one of dopamine. Even if it is not at the same potential, it could potentially interfere with the signal of dopamine (Fig S10a). However, for the closed device, the peaks of UA and AA slightly shift to a more negative potential, overlapping the peak of dopamine, as shown in Fig S10b. After the addition of Nafion the signal of the two interfering species have been obviously eliminated (Fig S10c). To study if the oxygen plasma treatment affects the effect of Nafion, the same concentrations of analytes were tested on devices treated with Nafion and afterwards with plasma. Fig S10c proves that Nafion is still able to inhibit the signal of the interference, even after oxygen plasma treatment. Because Nafion creates a very hydrophobic layer, it has to be diluted to allow the penetration of the solution inside the electrodes. As can be seen in Fig S10d, dilution of Nafion results in greater accessibility to porous LCNF electrode indicated by the higher magnitude of the signal in comparison to undiluted Nafion solution. Moreover, even if Nafion is diluted and plasma treated, its function in preventing signal interference from UA and AA remains the same.

**References**

1. Wongkaew N, Simsek M, Arumugam P, et al (2019) A Robust strategy enabling addressable porous 3D carbon-based functional nanomaterials in miniaturized systems. Nanoscale. 11:3674-3680. https://doi.org/10.1039/c8nr09232j

2. Lee H, Kharaghani D, Kim IS (2018) Mechanical Force for Fabricating Nanofiber. In: Lin T (ed) Novel Aspects of Nanofibers. IntechOpen, Rijeka

3. Wan LY, Wang H, Gao W, Ko F (2015) An analysis of the tensile properties of nanofiber mats. Polymer (Guildf) 73:62–67. https://doi.org/10.1016/j.polymer.2015.07.018
